# Correction: The anti-arthritis effect of sulforaphane, an activator of Nrf2, is associated with inhibition of both B cell differentiation and the production of inflammatory cytokines

**DOI:** 10.1371/journal.pone.0256716

**Published:** 2021-08-19

**Authors:** Su-Jin Moon, Jooyeon Jhun, Jaeyoon Ryu, Ji ye Kwon, Se-Young Kim, KyoungAh Jung, Mi-La Cho, Jun-Ki Min

There are errors in the Funding statement. The correct Funding statement is as follows: This study was supported by a grant of the Korean Health Technology R&D Project, Ministry for Health & Welfare, Republic of Korea (HI14C1851) and the National Research Foundation of Korea (NRF) grant funded by the Korea government (MSIT) (No. NRF-2018R1A2B6007648 and 2020R1I1A1A01072520).
