# Supplementary material for: The anti-arthritis effect of sulforaphane, an activator of Nrf2, is associated with inhibition of both B cell differentiation and the production of inflammatory cytokines
Source: PLoS One. 2021 Feb 16;16(2):e0245986. doi: 10.1371/journal.pone.0245986 (PMC7886167; doi:10.1371/journal.pone.0245986)
Supplement: S1 Fig — A,B. Reduction in arthritis score in CIA mice treated with Sulforaphane. CIA mice were injected intraperitoneal injected with sulforaphane (1.5mM, 12.8mg/mL/kg) every other day 7 weeks. (DOCX) [file pone.0245986.s001.docx]

**The anti-arthritis effect of sulforaphane, an activator of Nrf2, is associated with inhibition of both B cell differentiation and the production of inflammatory cytokines.**

**Su-Jin Moon^1*^, Jooyeon Jhun^2*^, Jaeyoon Ryu^2^, Ji ye Kwon^2^, Se-Young Kim^2^, KyoungAh Jung^3^, Mi-La Cho^2, 3, 4**^ and Jun-Ki Min^5**^**

^1^ Division of Rheumatology, Department of Internal Medicine, Uijeongbu St. Mary's Hospital, College of Medicine, The Catholic University of Korea, Uijeongbu, South Korea

^2^ The Rheumatism Research Center, Catholic Research Institute of Medical Science, The Catholic University of Korea, Seoul, South Korea

^3^ Impact Biotech, Seoul, 137-040, South Korea

^4^ Laboratory of Immune Network, Conversant Research Consortium in Immunologic disease, College of Medicine, The Catholic University of Korea

^5^ Department of Internal Medicine, and the clinical Medicine Research Institute of Bucheon St. Mary’s Hospital, Bucheon‑si, South Korea

**Authorship notes**

*****These authors contributed equally to this work

******These authors contributed equally to this work

**Address Correspondence to**

**Jun-Ki Min, MD, PhD,** Department of Internal Medicine, and the clinical Medicine Research Institute of Bucheon St. Mary’s Hospital, 327, Sosa-ro, Bucheon‑si, Gyeonggi-do, South Korea **(**Tel: 82-32-340-2013, Fax: 82-2-2258-7473, E-mail: rmin6403@hanmail.net)

**Mi-La Cho, PhD,** The Rheumatism Research Center, Catholic Research Institute of Medical Science, The Catholic University of Korea, 222 Banpo-Daero, Seocho-gu, Seoul, 137-701, South Korea (Tel: 82-2-2258-7467, Fax: 82-2-599-4287, E-mail: iammila@catholic.ac.kr)





**Figure S1: Sulforaphane-mediated inhibition of the development of CIA (A,B).**

A,B. Reduction in arthritis score in CIA mice treated with Sulforaphane. CIA mice were injected intraperitoneal injected with sulforaphane (1.5mM, 12.8mg/mL/kg) every other day 7weeks.
