# Supplementary material for: The anti-arthritis effect of sulforaphane, an activator of Nrf2, is associated with inhibition of both B cell differentiation and the production of inflammatory cytokines
Source: PLoS One. 2021 Feb 16;16(2):e0245986. doi: 10.1371/journal.pone.0245986 (PMC7886167; doi:10.1371/journal.pone.0245986)
Supplement: Attachment — Submitted filename: Figure 3 (Comments).pdf [file pone.0245986.s002.pdf]

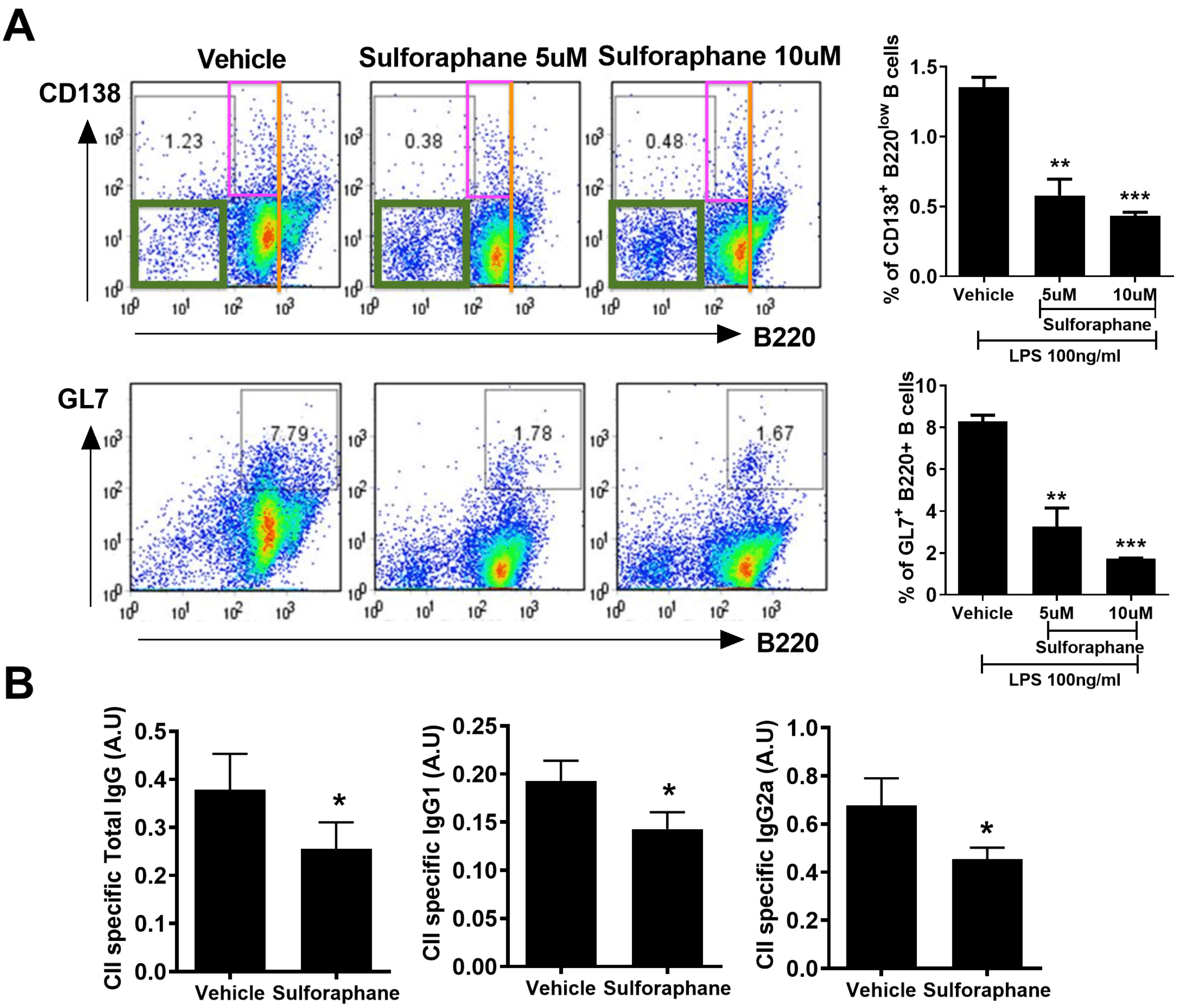

A
Vehicle
Sulforaphane 5uM
Sulforaphane 10uM
CD138
1.23
0.38
0.48
B220
GL7
7.79
1.78
1.67
B220
% of CD138+ B220low B cells
Vehicle
5uM
10uM
Sulforaphane
LPS 100ng/ml
% of GL7+ B220+ B cells
Vehicle
5uM
10uM
Sulforaphane
LPS 100ng/ml
B
CII specific Total IgG (A.U)
Vehicle Sulforaphane
CII specific IgG1 (A.U)
Vehicle Sulforaphane
CII specific IgG2a (A.U)
Vehicle Sulforaphane
