# Supplementary material for: The anti-arthritis effect of sulforaphane, an activator of Nrf2, is associated with inhibition of both B cell differentiation and the production of inflammatory cytokines
Source: PLoS One. 2021 Feb 16;16(2):e0245986. doi: 10.1371/journal.pone.0245986 (PMC7886167; doi:10.1371/journal.pone.0245986)
Supplement: Attachment — Submitted filename: Response to peer-reviewers_PLoS ONE.docx [file pone.0245986.s004.docx]

**Revised submission of manuscript PONE-D-20-25189**

Dear **David Douglass Brand**

Editor-in-PLOS ONE

We thank the reviewers for their constructive and helpful comments concerning the manuscript. We have addressed the reviewers' concerns by either performing additional statistical analysis and revising the manuscript or explaining respectfully our rebuttal. The point-by-point replies are given in this letter. We hope that we have addressed satisfactorily all concerns raised by the reviewers, and that this manuscript is now suitable for publication.

Thank you again for the comments.

Sincerely yours,

Mi-La Cho, PhD

Department of Medical Lifescience, College of Medicine, The Catholic University of Korea, 222, Banpo-daero, Seocho-gu, Seoul, 06591, Republic of Korea

(Tel: 82 2 2258 7467, Fax: 82 2 2258 7473, E-mail address: iammila@catholic.ac.kr)

**Comments from the editors and reviewers:**

Reviewer #1: The anti-arthritis effect of sulforaphane, an activator of Nrf2, is associated with inhibition of B cell differentiation

The manuscript investigates the effect of sulforaphane on collagen-induced arthritis. Mice are treated with sulforaphane intraperitoneally and the investigators demonstrate that there is a decrease in severity of arthritis. This is associated with decreases in histologic inflammation, decreases in cytokines IL-6, IL-17, TNF-a, and TRAP. The investigators also demonstrate that human PBMs can have inhibition in production of IL-6, TNF-a, and IL-17 when cultures with sulforaphane. This is an important finding and may have therapeutic implications.

**Answer:** We do appreciate the valuable comments from the reviewers about the manuscript. We have gone through the suggestive comments from all the reviewers and incorporated in the final revised version.

However, there are some problems with the manuscript.

The title is misleading. The effects of sulforaphane appear to be broad and cause inhibition of both T cell cytokines, as well as antibody production and inhibition of differentiation into plasma cells. Therefore, the focus on B cells is misleading.

**Answer:** Thanks for valuable comments. The authors agree with your comments and revised as you required. The title was revised as follows. “The anti-arthritis effect of sulforaphane, an activator of Nrf2, is associated with inhibition of both B cell differentiation and the production of inflammatory cytokines.”

There are really no experiments in the manuscript documenting that sulforaphane is actually an activator of Nrf2.

**Answer:** Thanks for your comments. We have no experiments in the manuscript. But, many

studies have shown that Sulforaphane is a potent activator of the endogenous anti-oxidant

transcription factor nuclear factor (erythroid-derived 2)-like 2 (Nrf2).

Reference

1. Eri Kubo, Bhavana Chhunchha, Prerna Singh, Hiroshi Sasaki & Dhirendra P. Singh. Sulforaphane reactivates cellular antioxidant defense by inducing Nrf2/ARE/Prdx6 activity during aging and oxidative stress. Scientific Reports, 7:14130 (2017)

2. Albena T. Dinkova-Kostova, Jed W. Fahey and Thomas W. Kensler. KEAP1 and done? Targeting the NRF2 pathway with sulforaphane. Trends in Food Science & Technology, 69:257-269(2017)

3. Christine A. Houghton, Robert G. Fassett, and Jeff S. Coombes. Sulforaphane and Other Nutrigenomic Nrf2 Activators: Can the Clinician’s Expectation Be Matched by the Reality? Oxidative Medicine and Cellular Longevity, 7857186 (2016)

There was no clear indication as to how many mice were used in the experiments described in figure 1A. It is important to know the number of mice in order to evaluate the validity of the results. For the flow experiments, it is also important to know how many mice were evaluated and what are the means of the populations evaluated.

Answer: The authors agree with your comments and revised as you required. We added the method section. (marked by red color, Page_5_, line 17_). We analyzed the experiments 3 times and showed the most representative data. We provided all the arthritis score graphs. These graphs are shown below.

Reviewer #2: This paper shows that sulforophan, known as an inhibitor of Nrf2, suppress collagen induced arthritis. It is claimed it do so based on its effects on B cells.

Answer: We really appreciate your time and effort to edit our manuscript. In this revised manuscript, we have resolved most of the issues raised by the reviewers as you can see in our response to their comments below.

Major points:
1) All data are not shown. Its not acceptable to base a study on an arthritis experiment with n=5. Apparently the experiment has ben run three times so this is a selected experiment. If the same experiment has been done 3 times it should be pooled and calculated together. The pooled data can be shown in the paper and the single experiments in the suppl information.

Answer: Thanks for valuable comment which can clarify the method of present study. The experiment was performed three times. We analyzed the experiments 3 times and showed the most representative data. As the trend of the experiment was consistent, it was shown as a representative picture.

2) It should be clearly stated that the experiment was done blindly and distributed in the cages randomly, especially as it is well known there is a strong cage effect in DBA/1 mice. It should also be stated that all animal experiments follow the ARRIVE guideline. Of course only if this was the case.

Answer: Thanks for comments. We performed grouping prior to Sulforaphane administration. To proceed with group separation, arthritis score and serum CII specific IgG2a measurement were analyzed. Then, group divide was conducted by ranking the results of two analyzes of arthritis score and CII specific IgG2a.

All experimental procedures were evaluated and conducted in accordance with the protocols approved by the Animal Research Ethics Committee at the Catholic University of Korea (Permit Number: CUMC 2016-0086-01). All procedures performed in this study followed the ethical guidelines for animal use.

3) The treatment has a profound effect on the inflammatory response. It is likely to have very broad effects and it will be difficult to say exactly what is the specific effect. Basically all readouts are secondary effects to something that this high dose of sulforophan is dong, whatever that is. Thus, it is not possible to claim that the effect on arthritis is due to effects on B cells as there is no evidence for this. The treatment is given 3 weeks after priming which means that the B cells have been primed and a full antibody response been developed. What will happen is that if these mice, due to this unknown "toxic" effect of the treatment does not develop arthritis it will secondarily, dur to less exposure of inflamed cartilage as well as a less powerful immune system give lower antibody titres. It can be predicted whatever is given to a mice leading to such a suppression of arthritis development.
In conclusion. If the arthritis data holds its a valuable report. But the authors need to make it very clear that they cannot say anything about the specific effects about sulforophan action as all evidenced data are secondary to the arthritis effect perse. Regarding human cells it seems to have profound inhibitory effect on cytokine production and I am afraid that these cells are not very happy.

Answer: Thanks for comments. We agree with the referee’s concern. As pointed out by the reviewers, there is insufficient direct evidence for inducing the immunomodulatory effect of B cells in the animal model of rheumatoid arthritis as a direct response to the drug. Antioxidants such as sulforafan can appear when excessive inhibition of oxidative stress is accompanied by an inflammatory response. Therefore, when using antioxidant drugs, dosages that do not have cellular toxic effects and inflammatory responses should be used. However, there seems to be a difficulty because the dosage used for the drug is not proportional even between in vitro and in vivo experiments. As a reviewer's opinion, it seems difficult to identify directly in vivo that the therapeutic effect in an animal model is a direct regulatory response to B cells from our results. In the future, we will take this part into account and try to confirm it in subsequent studies. Thank you very much for your review comment.
